# Combination of paeoniflorin and liquiritin alleviates neuropathic pain by lipid metabolism and calcium signaling coordination

**DOI:** 10.3389/fphar.2022.944386

**Published:** 2022-09-07

**Authors:** Yan-Yan Chen, Li-Mei Feng, Ding-Qiao Xu, Shi-Jun Yue, Rui-Jia Fu, Mei-Mei Zhang, Yu-Ping Tang

**Affiliations:** Key Laboratory of Shaanxi Administration of Traditional Chinese Medicine for TCM Compatibility, and State Key Laboratory of Research & Development of Characteristic Qin Medicine Resources (Cultivation), Shaanxi Key Laboratory of Chinese Medicine Fundamentals and New Drugs Research, and Shaanxi Collaborative Innovation Center of Chinese Medicinal Resources Industrialization, and Shaanxi University of Chinese Medicine, Xi’an, Shaanxi, China

**Keywords:** paeoniflorin, liquiritin, neuropathic pain, lipid metabolism, calcium signaling pathway

## Abstract

Neuropathic pain (NP) affects 7%–10% of the general population and is still hard to cure. Here, we validated the therapeutic effect and demonstrated the mechanism of paeoniflorin and liquiritin combination (PL) on NP from the perspective of integrated lipidomics and transcriptomics for the first time. SwissTargetPrediction indicated that PL mainly targets lipid metabolism. Notably, lipidomics revealed that imbalanced lipid levels in the NP model could be reprogrammed to normal levels by PL treatment. RNA-sequencing showed that PL treatment could also rebalance the lipid metabolism in an indirect manner. Pathway analysis highly enriched the calcium signaling pathway among the most significant categories. Altogether, these findings suggested that PL can not only balance the lipid metabolism in direct and indirect manners but also reverse the dysfunctional activation of the calcium signaling pathway, thereby alleviating NP. This helps to better understand the mechanisms of NP and provides a new important potential therapeutic option for NP.

## Introduction

Neuropathic pain (NP) is a common chronic pain condition caused by a lesion or disease of the somatosensory nervous system that affects 7%–10% of the general population (Finnerup et al., 2021). Currently, the management of neuropathic pain includes pharmacological, interventional, and physical therapy. Among them, pharmacotherapy remains an important modality, with calcium channel α2-δ ligands such as pregabalin, serotonin-noradrenaline reuptake inhibitors such as duloxetine, and various tricyclic antidepressants recommended as first-line treatment for neuropathic pain (Colloca et al., 2017). However, the limited efficacy and intolerable side effects of these drugs suggest a huge unmet medical need for drug development.

Natural products have become a priority strategy for the treatment of various disorders including NP. Paeoniflorin, the main bioactive component of Paeoniae Radix Alba (Shaoyao), has been reported to exert analgesic effect on NP and inflammatory pain (Yin et al., 2016; Hu et al., 2018; Zhou et al., 2019). Liquiritin, an active ingredient derived from Glycyrrhizae Radix et Rhizoma (Gancao), exhibited anti-neuropathic action due to its protective effect on damaged nerves and its anti-inflammatory activity at the level of the spinal cord (Zhang et al., 2017). Cocktail therapy has emerged as a promising way to overcome the clinical challenge (Chaparro et al., 2012). Moreover, Paeoniflorin and liquiritin has been reported as a potential combination to alleviate NP by inhibiting neuroinflammation (Guo et al., 2020). Although PL has been indicated to alleviate NP, the systemic effect and underlying mechanism of PL-mediated alleviation still remain exclusive.

Integration of multiple omics technologies has emerged as an approach to provide a comprehensive view of health and disease and may provide a better understanding of pharmacotherapy (Karczewski and Snyder, 2018). The lipids (i.e., sphingolipids, ceramides, and lysophosphatidic acids) are critical for the initiation of NP (Patti et al., 2012; Langeslag and Kress, 2020), which called for the application of lipidomics, a branch of metabolomics that focuses on lipids and analyzes lipid metabolism network changes under different physiological conditions (Yang and Han, 2016). Transcriptomics is mainly performed to identify the gene expression profile which could provide an insight into the alteration of biological processes at the gene level. The integration of lipidomics and transcriptomics analysis may provide higher reliability in illustrating the action and mechanism.

In the current study, leveraging on drug potential target prediction, RNA-sequencing, and lipidomics, we revealed PL may alleviate NP by reprogramming unbalanced lipid metabolism in both direct and indirect manners and inhibiting the calcium signaling pathway.

## Materials and methods

### Chemicals and reagents

Paeoniflorin (C23H28O11, MW: 480.5, purity >98%) and liquiritin (C21H22O9, MW: 418.4, purity >98%), the main active components of *Paeonia lactiflora* Pall. and *Glycyrrhiza uralensis* Fisch. ex DC., respectively, were purchased from Nanjing Yuanzhi Biotechnology Co., Ltd. (Nanjing, China). LC-MS grade methanol, acetonitrile, formic acid, and isopropanol were purchased from Fisher Scientific (Waltham, MA, United States). HPLC grade methyl tert butyl ether (MTBE) was obtained from Fisher Scientific (Waltham, MA, United States). The standard lipids were obtained from Avanti Polar Lipids (United States). Deionized water was purified using a Milli-Q system (Millipore, Bedford, MA, United States). All other chemicals and reagents used in this study were of analytical grade.

### Animals and treatment

Animals: Male Sprague Dawley rats (*n* = 48, 180–200 g) were purchased from Chengdu Dossy Experimental Animals Co., Ltd. (Chengdu, China) with license No. Of SCXK 2020-030. All animals were kept in standard animal houses with regulated temperature (20–25°C), humidity (40%–60%), and a 12-h light/dark cycle and were fed with food and water *ad libitum* before the experiment. The experimental procedures were in accordance with the animal care and ethical guidelines for the experimental investigation of pain in conscious animals of the National Institutes of Health.

SNI model establishment: To establish the spared nerve injury (SNI) model, the rats were anesthetized with 30 mg/kg pentobarbital sodium administered intraperitoneally and placed in a prone position. An incision was made on the lateral surface of the thigh, and three branches of the sciatic nerve (the sural, common peroneal, and tibial nerves) were exposed. Both common peroneal and tibial nerves were ligated together with 5.0 silk and transected approximately 2 mm distal to the ligation, while the sural nerve was left intact. The muscles and skin were sewed up with 5.0 silk sutures (Decosterd and Woolf, 2000). Sham-operated rats underwent the same procedure except ligation and transection of the nerves.

Drug administration: 48 rats were randomly divided into six groups (*n* = 8): the sham-operated (JA) group, spared nerve injury (SNI) group, PLL group (SNI +10.33 mg/ml paeoniflorin and 1.99 mg/ml liquiritin, 1 ml solution per 100 g), PLM group (SNI + 20.65 mg/ml paeoniflorin and 3.98 mg/ml liquiritin), PLH group (41.30 mg/ml paeoniflorin and 7.96 mg/ml liquiritin), and positive (PGB) group (SNI + 15 mg/kg pregabalin). The dose of PLM group was equal to the actual content of those components in the Shaoyao Gancao Decoction. Gastric infusion was performed continuously for 21 days after operation. Rats in the treatment groups were given 1 ml solution per 100 g, and rats in the JA group and SNI group only received the same volume of saline.

### Behavioral assessment

To test the effect of paeoniflorin and liquiritin combination (PL) for alleviating NP, the mechanical withdrawal threshold (MWT) was measured 1 day before and 1, 3, 5, 7, 9, 11, 14, 17, and 21 days after SNI. Rats were individually placed in a test cage on a metal grid, and after 30 min of adaptation, MWT was measured at the lateral side of the surgical paw by detecting the hind paw withdrawal responses to Von Frey filaments (North Coast Medical Company, United States) under pressure of 0.6–15.0 g (0.6, 1.0, 1.4, 2.0, 4.0, 6.0, 8.0, and 15.0 g). We started with an intermediate filament (2.0 g) perpendicular to the fourth and fifth toes (sural innervation area) on the hind paw surface and bent the filament slightly for about 6–8 s with sufficient force. If the response was positive (claw retracts rapidly in 3 s), “X” was recorded and the next weaker filament was used. If the response was negative, “O” was recorded and the next stronger filament was used. The interval time of each stimulation should be more than 2 min. When the combination of “XO” or “OX” appears, a series of “O” or “X” combination sequences should be obtained by stimulating another four times. The formula MWT (g) = (10^ [xf + k*δ])/10,000, where xf is the logarithm of the last filament in the sequence, k is the corresponding value of the sequence in the k-value table, and δ is the average difference after log for the strength of each filament. If “O” appears for five consecutive times, it is recorded as 15.0 g; if “O” occurs for five consecutive times, it is recorded as 0.6 g. If calculated MWT >15.0 g or <0.6 g, then 15.0 g or 0.6 g is still taken as the maximum or minimum value (Chaplan et al., 1994).

### Immunofluorescence assay

Astrocytes and microglia have recently emerged as key contributors to the pathology of NP (Echeverry et al., 2017). Glial fibrillary acidic protein (GFAP) and ionized calcium binding adapter molecule 1 (IBA-1) expression were used as markers of astrocyte and microglia activity, respectively. On the 21st day, the rats were anesthetized with 30 mg/kg pentobarbital sodium administered intraperitoneally and terminated; L3-L6 of the spinal cord were collected for immunofluorescence (IF), and spinal cord sections were washed with phosphate-buffered saline (PBS) 3 times and exposed to 0.5% bovine serum albumin (BSA) for 1 h at room temperature. Then, sections were incubated with primary antibodies of anti-GFAP mouse polyclonal antibody (1:400, CST, United States) for detecting astrocytes and anti-IBA-1 rabbit monoclonal antibody (1:400, Abcam, United States) for detecting microglia overnight at 4°C. After washing with PBS, sections were incubated with secondary antibodies of goat anti-mouse antibody (1:200, Servicebio, China) against GFAP and donkey anti-rabbit antibody (1:200, Servicebio, China) against IBA-1 for 1 h at room temperature. Next, the glass slides were covered with 4,6-diamidino-2-phenylindole (DAPI) for 5 min at room temperature and washed 3 times with PBS. After coverslips were placed, the labeled sections were immediately observed under a fluorescence microscope (Nikon Eclipse C1, Japan). Image Pro Plus 6.0 was used to analyze optical densities. The intensity of GFAP and IBA-1 expression was quantified by integrated optical density (IOD) values obtained from the threshold pixels for all signals measured in each image and corresponding pixel area (Area). For each section, three representative visual fields in the ipsilateral dorsal horn were chosen to calculate the areal density value; area density = IOD/Area.

### Western blot analysis

Briefly, total protein extract was prepared by homogenizing the ipsilateral L3-L6 spinal cord tissues using RIPA lysis buffer and protease inhibitor cocktail. The protein concentration was measured using the BCA Protein Assay Kit. Equal amount of each protein was separated on SDS-PAGE gels and transferred to PVDF membranes. After blocking with 5% BSA at room temperature for 1 h, the membranes were incubated at 4°C overnight with the appropriate primary antibody: GFAP, IBA-1, or β-actin (4970, CST, United States). The membranes were then washed and incubated with horseradish peroxidase (HRP) conjugated secondary antibodies (ab7097, Abcam, United States) for 1.5 h at room temperature. Reactive bands were visualized using the super ECL plus kit and captured using a gel imaging system.

### Quantitative RT-PCR

Total RNA was extracted using Trizol reagent (Invitrogen), and the cDNA was obtained using the PrimeScriptTM RT Reagent Kit (TaKaRa) according to the manufacturer’s protocol. The relative mRNA levels of target genes were measured by quantitative RT-PCR analyses using the SYBR Green Prime Script kit (Bio-Rad). The primers for all genes are shown below. Hrh1, F: CTGGCTCGGGGAGGGAG, R: CTG​TCC​TGT​TCC​CCT​CAC​AC; Tacr1, F: CGT​ACA​CTG​TGG​TGG​GGA​TTA, R: ATC​ATT​TTG​ACC​ACC​TTG​CGT; Adora2b, F: ATC​TTT​AGC​CTC​TTG​GCG​GT, R: ACC​AAA​CCT​TTA​TAC​CTG​AGC​G; Ednrb, F: CCA​ATC​CGT​GCG​AGA​CTG​AA; R: ATA​CCC​CCA​ACA​AGC​CAC​AG; Agt, F: CTC​AGG​CCA​AGC​TGT​CTA​CC, R: GCT​GTT​GAG​AAC​CTC​TCC​CA.

### Lipidomics

Serum samples: Blood was harvested *via* retro-orbital plexus into tubes on the 21st day and centrifuged at 13,000 rpm for 10 min at 4°C. Serum samples were separated and stored at −80°C until analysis. Each 40 µL serum sample was mixed with 300 µL methanol to precipitate proteins and homogenized with 1 ml MTBE for 1 h to extract lipid, followed by the addition of 250 µL H_2_O to induce phase separation. The resulting two-phase solution was set at room temperature for 10 min and centrifuged at 13,000 rpm for 10 min at 4°C. Subsequently, a 400-µL MTBE layer was separated into a new vial and dried using a SpeedVac. The dried upper layer was reconstituted in 100 µL solution (97 µL isopropanol/acetonitrile with a volume ratio of 1:1, 3 μL PC (12:0/13:0) with a concentration of 15.16 μmol/L as the internal standard solution), centrifuged at 13,000 rpm for 10 min at 4°C, and the supernatant was used for lipidomics analysis.

Tissue samples: Each ipsilateral side of L3-L6 spinal cord samples were weighed and homogenized in a homogenizing tube and mixed with 300 µL methanol and 1 ml MTBE for 1h, followed by adding 250 µL H_2_O to induce the phase separation. A 400-µL MTBE upper layer was separated and dried and then reconstituted in 200 µL solution (194 µL isopropanol/acetonitrile with a volume ratio of 1:1, 6 μL PC (12:0/13:0) with a concentration of 15.16 μmol/L) centrifuged at 13,000 rpm for 10 min; the supernatant was used for lipidomics analysis.

UPLC-TQ-MS analysis: Processed serum or spinal cord samples were subjected to chromatographic separations on a Waters UPLC BEH C18 column (2.1 × 100 mm, 1.7 µm) using an UPLC I-Class system (Waters Corporation, United States). The mobile phase consisted of A (a mixture of acetonitrile and water (6:4) with 0.1% formic acid and 5 mM ammonium acetate) and B (a mixture of isopropanol/acetonitrile (9:1) with 0.1% formic acid and 5 mM ammonium acetate). The following gradient elution was employed at a flow rate of 0.26 ml/min: 32% B at 0–1.5 min, 32–85% B at 1.5–15.5 min, 85–97% B at 15.5–15.6 min, 97% B at 15.6–18.0 min, 97%–32% B at 18.0–18.1 min, and 32% B at 18.1–20.0 min. The column temperature was maintained at 4°C and the injection volume was 5 μL. Detection of the separations was achieved using a triple quadrupole mass spectrometer (TQ-MS) system (Waters Corporation, UK) operated in positive ion mode. The source temperature was 150°C with a capillary voltage of 3.0 KV and a sampling cone voltage of 30 V. The desolvation gas temperature was set at 500°C with a cone gas flow of 50 L/h, and the desolvation gas flow was 1000 L/h. High-purity argon was employed as collision gas, the collision gas flow rate was 0.15 ml/min, and high-purity nitrogen was used in other gas paths. In order to obtain reliable results and improve the accuracy of lipid analysis, a target database including 115 phosphatidylcholines (PCs), 30 lysophosphatidylcholines (LPCs), 28 phosphatidylethanolamine (PEs), 4 lysophosphatidyl ethanolamine (LPEs), 8 ceramides (Cers), 42 sphingomyelins (SMs), 75 triglycerides (TGs), and 4 choline ethanolamines (CEs) was constructed based on the LC-MS platform using internal standard standards as well as the relative data of accurate masses and MS/MS fragments of the lipids (Supplementary Table S1).

Data processing: Skyline Software (https://skyline.gs.washington.edu/labkey/project/home/software/Skyline/begin.view) was used to create lipid transitions, visualize results, integrate observed signals, and quantify all lipids that were detected by MS. For each target lipid, precursor product ion pairs (transitions) were selected, and the corresponding peak area of each transition was calculated using software. The ion tolerance of the extraction peak was 0.05 Da. Then the relative quantification of the target metabolites was carried out by using the homologous internal lipid standard.

Data analysis: Multivariate analysis was performed using SIMCA P 14.1 software. An unsupervised model of principal component analysis (PCA) was used to evaluate the holistic metabolite alterations among groups and monitor the stability of this study. A supervised model of orthogonal partial least squares discrimination analysis (OPLS-DA) was performed to maximize the distance between groups and identify variables with important contribution to the classification according to the variable importance in projection (VIP) values. Metabolites that met the following criterion of adjusted *p*-values < 0.05, VIP >1.0, and fold change >2 were considered as differential metabolites.

### Transcriptomics

Sample preparation: Total RNA was isolated from the ipsilateral side of L3-L6 spinal cord tissues (JA group, SNI group, and PLM group) using the RNeasy Plus kit followed by a genomic DNA removal step. The concentration and quality of extracted RNA were estimated using a NanoDrop2000 spectrophotometer (Thermo Fisher Scientific, United States), the integrity of RNA was detected by agarose gel electrophoresis, and the RNA integrity number (RIN) was measured using an Agilent 2100 bioanalyzer (Agilent Technologies, Inc., United States).

RNA-Sequencing: A total of 3 μg RNA per sample was utilized as input material. The Illumina TruseqTM RNA sample prep Kit was used to generate sequencing libraries. Illumina sequencing by synthesis technology was applied to perform the sequence of the resultant double-stranded Complementary DNA (cDNA) libraries. The raw sequencing reads were cleaned by removing adaptors and low-quality reads (reads with a Q-value < 20). Clean reads were mapped to the reference genome using HISAT2 v2.0 with default parameters. Gene expression values of the transcripts were computed using StringTie v1.3.3b.

Data analysis: Differential expression analysis of two groups was performed using DESeq2 Version 1.12. The *p*-value < 0.05 and |log2FC| > 1 (FC, fold change) were chosen as the threshold for screening differentially expressed genes (DEGs). Functional enrichment analyses, including Kyoto Encyclopedia of Genes and Genomes (KEGG) pathway enrichment and Gene Ontology (GO) enrichment analysis, were performed using the R package. KEGG pathways and GO terms with false discovery rates *p* < 0.05 were considered as significantly altered.

### Integrated lipidomics and transcriptomics analysis

The datasets of lipidomics and transcriptomics were integrated using MetaboAnalyst 5.0 (https://www.metaboanalyst.ca) to examine the potential relationship between significantly changed metabolites and DEGs.

### Statistical analysis

All statistical evaluations of lipidomics data were calculated from relative abundances. Experimental data were presented as mean ± standard deviation (SD). Comparisons were made by unpaired two-tailed Student’s *t*-tests or one-way analysis of variance (ANOVA) using Graphpad Prism 8.0. *p* < 0.05 was considered statistically significant.

## Results

### Establishment of SNI model

**FIGURE 1:**
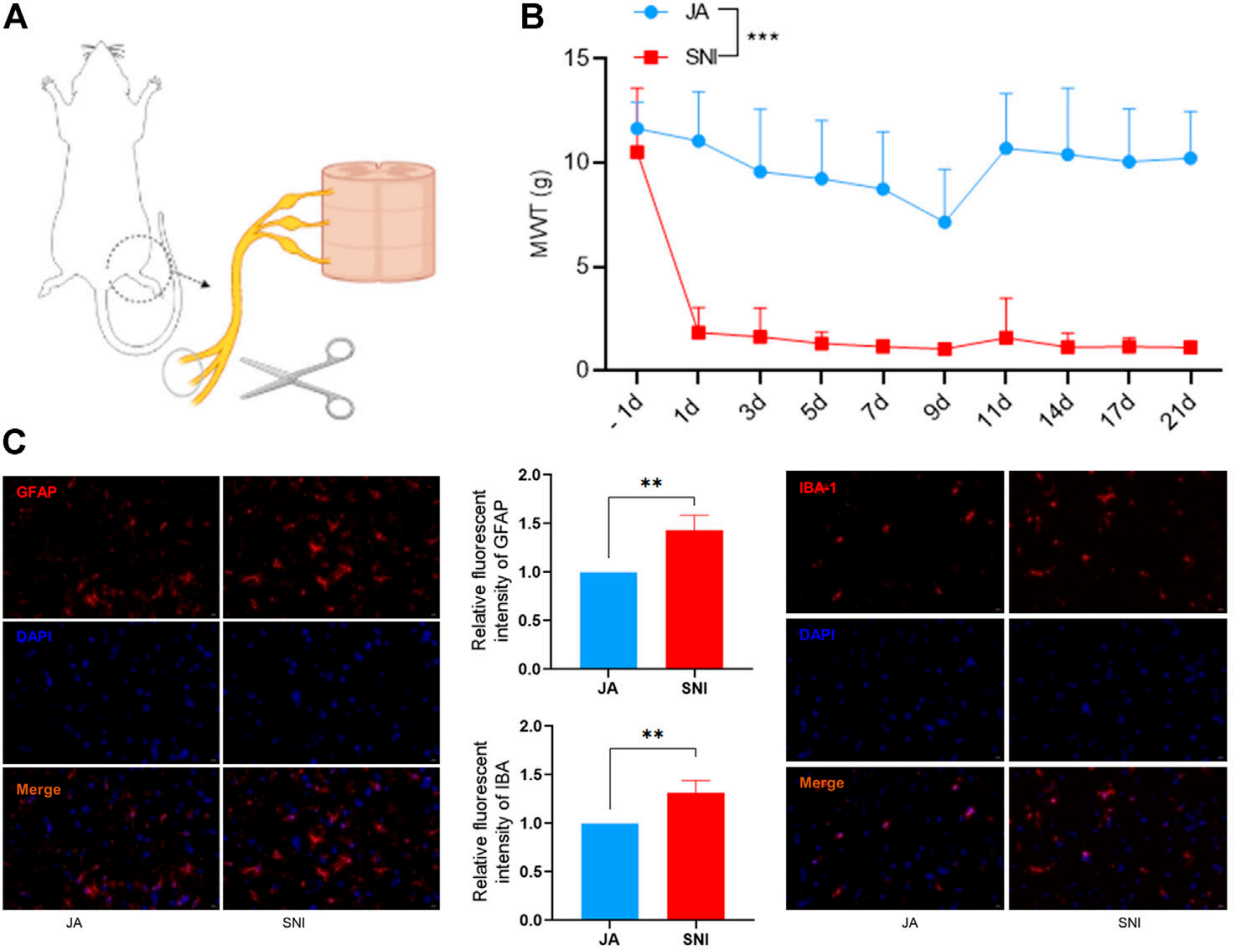
**(A)** Establishment of the SNI model. **(B)** Mechanical withdrawal threshold (MWT) of JA and SNI. **(C)** The expression of GFAP and IBA-1 in JA and SNI determined by immunofluorescence. JA, sham-operated group; SNI, spared nerve injury group. Compared with JA, ** *p* < 0.01, *** *p* < 0.001.

To investigate the role of PL treatment in NP, we established an SNI model which had been widely used to mimic physical damage to peripheral nerves. As shown in Figure 1A, we ligated and transected the common peroneal and tibial branches of the sciatic nerve which evoked intense mechanical allodynia (Decosterd and Woolf, 2000). MWT, a common marker for assessing NP, was strikingly increased in the SNI model compared to the JA group (Figure 1B). Moreover, a significant induction of GFAP and IBA-1 was also observed in the ipsilateral spinal dorsal horn of the SNI model compared to the JA group (Figure 1C). Collectively, these data indicated that the NP model has been successfully constructed.

### Combination of paeoniflorin and liquiritin alleviates NP

**FIGURE 2:**
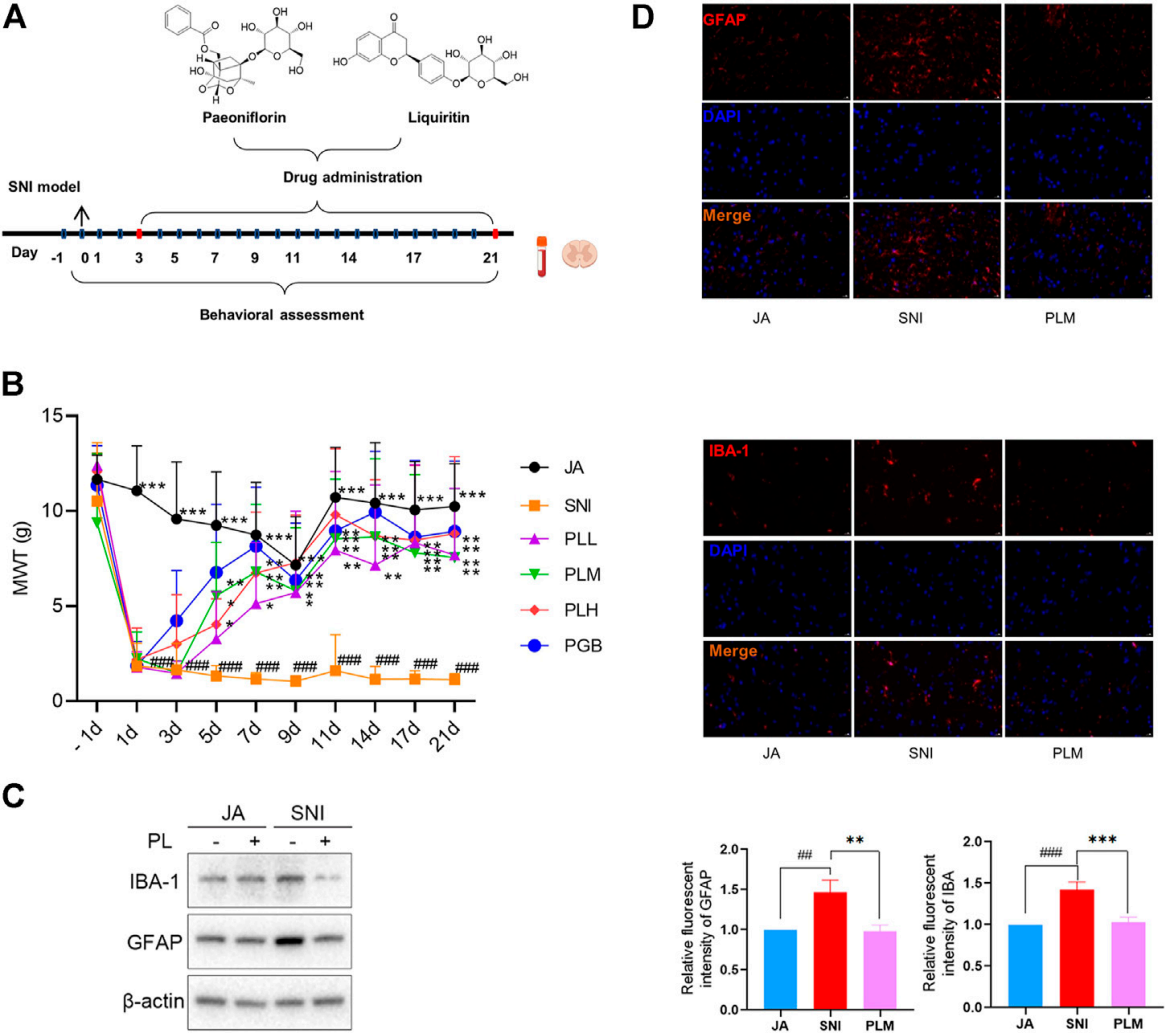
**(A)** Experimental roadmap of paeoniflorin and liquiritin combination on SNI. **(B)** Mechanical withdrawal threshold (MWT) of each group. **(C)** The expression of GFAP and IBA-1 in JA, SNI, and PLM evaluated by Western blot. **(D)** The expression of GFAP and IBA-1 in JA, SNI, and PLM evaluated by immunofluorescence. JA, sham-operated group; SNI, spared nerve injury group; PLL, PLM, and PLL, low, middle, and high dose of paeoniflorin and liquiritin combination group; PGB, pregabalin group. Compared with JA, ## *p* < 0.01, ### *p* < 0.001; compared with SNI, * *p* < 0.05, ** *p* < 0.01, *** *p* < 0.001.

To reveal the role of PL in NP, we treated the SNI rats with a gradient dose of PL or PGB which was set as a positive control (Figure 2A). Comparable to PGB, PL treatment significantly shortened the MWT (Figure 2B). Due to the medium dose of PL (PLM) achieving the preferable efficiency in alleviation of NP, this dose was used for further studies. In consistence with the MWT results, PLM also restored the GFPA and IBA-1 expression induced by ligation and transection operation (Figures 2C,D). Taken together, PL treatment exhibited a notable effect in alleviating NP.

### Paeoniflorin and liquiritin targets lipid metabolism

**FIGURE 3:**
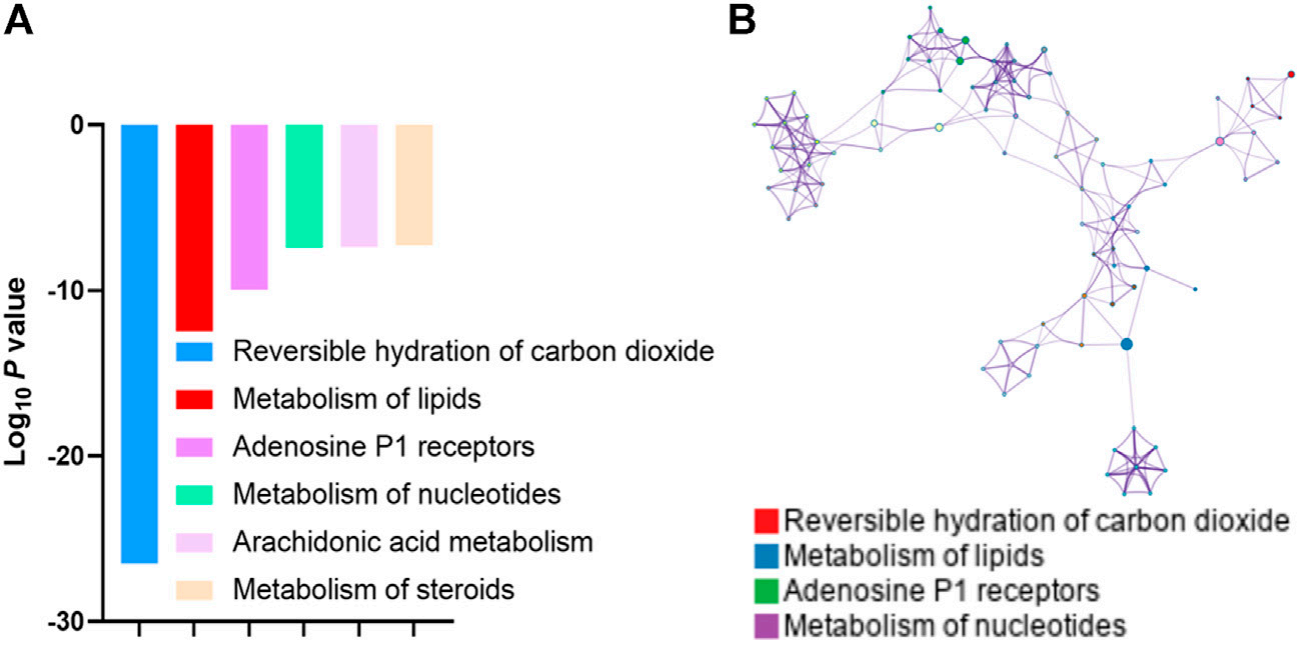
**(A)** Gene Ontology analysis of the potential targets of paeoniflorin and liquiritin predicted by SwissTargetPrediction. **(B)** GO pathway and process enrichment analysis of each component of the interacting network member of potential targets.

To gain more insights into the molecular mechanism on how PL treatment alleviated the NP, we used SwissTargetPrediction (http://swisstargetprediction.ch/) to dig out the potential targets of PL (Supplementary Table S2). Pathway analysis of these targets enriched “Reversible hydration of carbon dioxide” and “Lipid Metabolism” (Figure 3A). Meanwhile, pathway and process enrichment analysis has been applied to each component of the interacting network member of potential targets and “Lipid Metabolism” had also been suggested to be the most enriched one (Figure 3B). Accumulated evidence revealed that the imbalance of lipid metabolism led to initializing the NP. Accordingly, we hypothesized that PL may relieve the NP by reprogramming lipid metabolism.

### Combination of paeoniflorin and liquiritin reversed the lipid perturbations in SNI model

**FIGURE 4:**
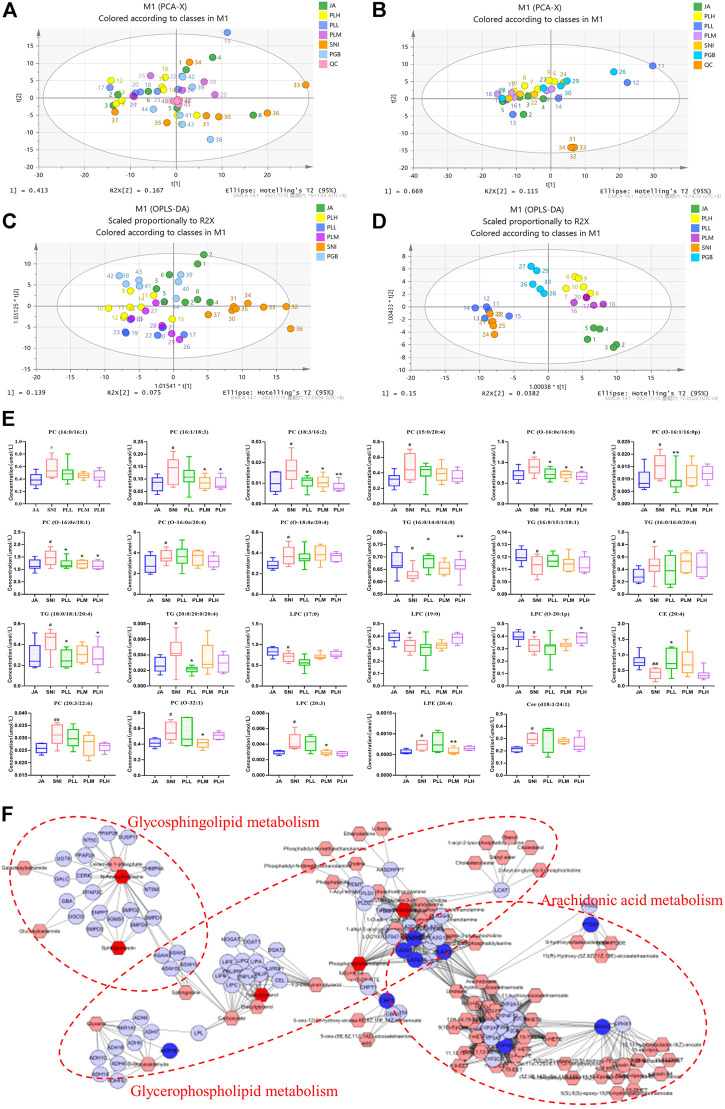
PCA plots of serum samples **(A)** and tissue samples **(B)**. OPLS-DA plots of serum samples **(C)** and tissue samples **(D)**. **(E)** Metabolic changes of lipids in the five test groups. **(F)** Integrated pathway analysis of potential targets and dysregulated lipid metabolites using MetScape. JA, sham-operated group; SNI, spared nerve injury group, PLL, PLM, and PLL, low, middle, and high dose of paeoniflorin and liquiritin combination group; PGB, pregabalin group; QC, quality control.

Since imbalanced lipid metabolism contributed to NP and PL had been indicated to mainly target the key regulators in the lipid metabolism process, we speculated dysregulated lipid metabolism may occur in the SNI model in comparison with the JA group and PL treatment could reprogram this process. To test this hypothesis, we first thought to gain a global view of the lipid profile in each group by lipidomics. The lipid metabolic phenotypes based on all imported samples could be classified by PCA. An overview of all samples in the data and a grouping trend between the JA group, SNI group, PLL group, PLM group, and PLH group could be observed. As shown in Figures 4A,B, the cluster of QC samples in the PCA score plot exhibited the stability and repeatability of this lipidomic analysis system. In the OPLS-DA model of the JA group and the SNI group, the cumulative R2Y and Q2 were 0.912 and 0.517 for serum samples, while that of tissue samples were 0.929 and 0.670, which showed that the model is reliable (Supplementary Figure S1). OPLS-DA was further employed for all samples, and a clear separation of the JA group, SNI group, PLL group, PLM group, PLH group, and PGB group was observed (Figures 4C,D). In consistence with the shorter MWT and restoration of GFAP and IBA, the PLM group was significantly closer to the JA group, indicating that the analgesic effect of the middle dose group was more significant. In-depth analysis of lipid metabolites based on VIP >1, *p* < 0.05 revealed that 18 specific differential metabolites in the SNI model were derived from serum samples, and five metabolites were identified from spinal cord samples (Figure 4E). In detail, the lipid levels of 11 PCs, 3 TGs, 1 LPC, 1 LPE, and 1 Cer were significantly upregulated while 2 TGs, 3 LPCs, and 1 CE were remarkably reduced in the SNI group compared with the JA group. Notably, PL treatment could re-balance the majority of these dysregulated metabolites (16/23) including PC (16:1/18:3), PC (18:3/16:2), PC (O-16:0e/16:0), PC (O-16:1/16:0p), PC (O-16:0e/18:1), PC (O-32:1), TG (16:0/14:0/16:0), TG (18:0/18:1/20:4), TG (20:0/20:0/20:4), LPC (O-20:1p), LPC (20:3), LPE (20:4), and CE (20:4) (Figure 4E). Next, the potential targets and dysregulated lipid metabolism pathway were aligned to gain an insight into the molecular mechanisms of reprogramming of lipid metabolism by PL. Surprisingly, the direct targets could only partially account for the restoration of imbalanced metabolites, such as glycosphingolipid metabolism, glycerophospholipid metabolism, and arachidonic acid metabolism, which highlighted the fact that PL might reprogram the lipid metabolism in both direct and indirect ways (Figure 4F). Taken together, PL alleviated NP through reprogramming lipid metabolism.

### Combination of paeoniflorin and liquiritin also balanced lipid metabolism in SNI model via an indirect manner

**FIGURE 5:**
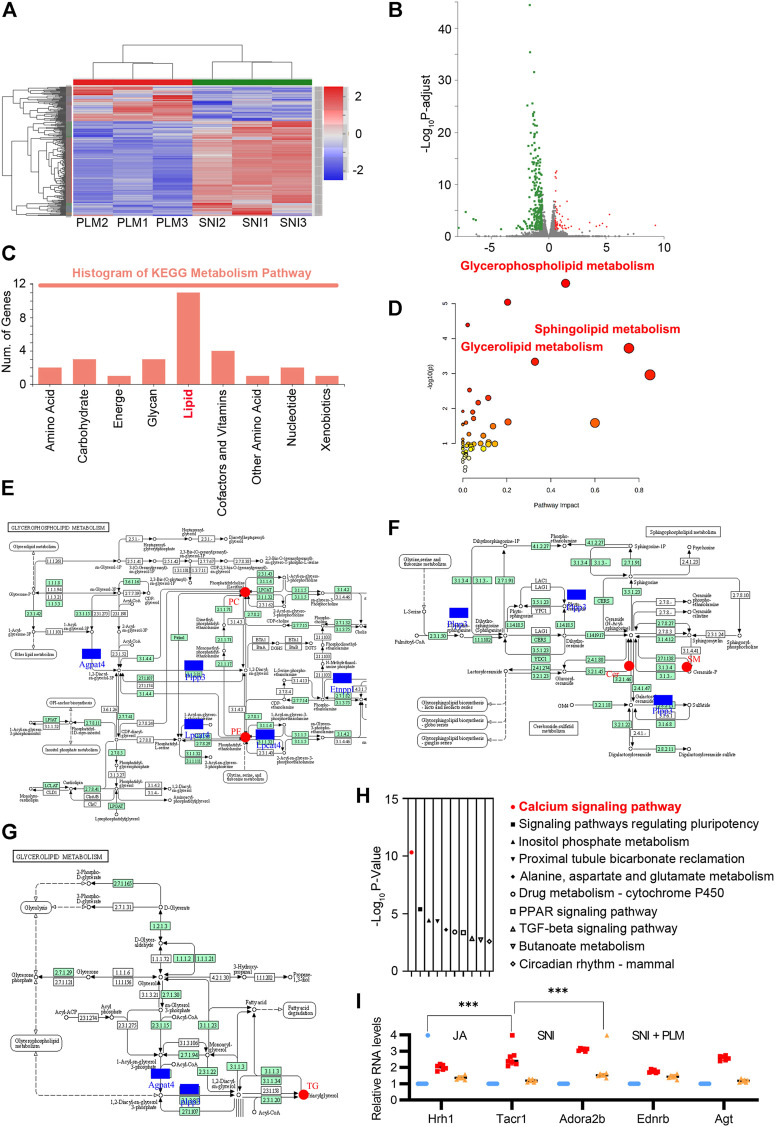
**(A)** Heatmap analysis of SNI and PLM in transcriptomic analysis (fold change >2). **(B)** Volcano map of the differentially expressed genes of SNI and PLM (fold change >2). **(C)** KEGG enrichment analysis of downregulated genes upon PL treatment. **(D–G)** Lipidomics-transcriptomics network built using MetPA. **(H)** GO enrichment analysis of downregulated genes upon PL treatment. **(I)** Relative mRNA expression of Hrh1, Tacr1, Adora2b, Ednrb, and Agt in the calcium signaling pathway detected by quantitative RT-PCR assays. SNI, spared nerve injury group; PLM, middle dose of paeoniflorin and liquiritin combination group.

To further characterize the molecular mechanism of the combination of paeoniflorin and liquiritin in regulation of the lipid metabolism and the alleviation of NP, we compared the gene expression profile in the SNI model with or without PLM treatment by RNA-sequencing. As shown in the heatmap, each independent sample was highly correlated (Figure 5A). A total of 556 downregulated and only 127 upregulated genes were identified in PLM in comparison with SNI (Figure 5B). Next, we performed GO analysis around the downregulated genes. Hence, we focused on the metabolic pathway and found the lipid metabolism pathway was most enriched in this analysis set which elaborated that PLM was involved in regulation of lipid metabolism in an indirect manner (Figure 5C). Then we integrated the RNA-seq data and lipidomics to delve into the correlation between PLM-regulated genes and alteration of lipid metabolites. The combined analysis identified significantly enriched biochemical pathways (*p* < 0.05 and impact >0.15), including glycerophospholipid metabolism, glycerolipid metabolism, sphingolipid metabolism, and ether lipid metabolism (Figure 5D). Consistent with our hypothesis, a lipidomics-transcriptomics network was built to screen the significantly changed lipids and genes located at key nodes (Figures 5E–G). Collectively, our data indicated PL reedited lipid metabolism to alleviate NP by directly targeting key nodal molecules of lipid metabolism and inhibiting transcription of genes important for lipid metabolism.

Notably, pathway analysis of these downregulated genes highly enriched “Calcium signaling pathway” among the most significant categories (Figure 5H). PL efficiently restored the increase of Hrh1, Tacr1, Adora2b, Ednrb, and Agt in the calcium signaling pathway caused by NP (Figure 5I). Due to accumulated evidence revealing that an aberrantly activated calcium signaling pathway could evoke the NP (Finnerup et al., 2021), our above analysis highlighted a pivotal role of the combination of paeoniflorin and liquiritin in relief of NP through not only balancing the lipid metabolism in both direct and indirect ways but also reversing the dysfunctional activation of the calcium signaling pathway.

## Disccusion

This study presents data to support the anti-NP potential mechanism of PL, the principal active constituents of Shaoyao Gancao decoction (a classical formula that originated in the Han Dynasty for pain relief). Although a few anti-NP mechanisms involved in paeoniflorin or liquiritin have been explored (Zhang et al., 2017; Zhou et al., 2019), and a biomolecular network–based study showed PL may alleviate NP by inhibiting neuroinflammation through reducing the expression and activity of the CCL5-CCR5-GNAI1-SRC-PIK3CA-AKT signal axis (Guo et al., 2020), these studies were mostly limited to exploring molecular targets in traditional methods or combined with network pharmacology, which was hardly to interpret the pharmacological mechanisms thoroughly. Taking advantage of the prospective characteristics of multi-omics integration, lipidomics and transcriptomics were employed to provide the systematic perturbation of PL on NP.

The role of lipids in NP has gained attention in recent years. It was shown that lipid metabolism such as neuropathic-ceramide metabolism was altered in the dorsal horn of rats with NP (Langeslag and Kress, 2020). The increase of PC (16:0/20:4) level in the dorsal root ganglion was associated with NP (Mihara et al., 2019). LPC (18:1) and LPC (16:0), as well as the linoleic acid metabolite, were significantly increased in the dorsal root ganglion and spinal cord tissue of peripheral NP animals as revealed by lipidomics (Rimola et al., 2020). In this study, UPLC-TQ-MS based lipidomics was employed to analyze eight classes of lipids. The increase in PC (16:1/18:3), PC (18:3/16:2), PC (O-16:0e/16:0), PC (O-16:1/16:0p), and PC (O-16:0e/18:1) and the decrease in LPC (O-20:1p) in serum sample were observed in the SNI group, and these lipids were adjusted to nearly normal levels by PL. Meanwhile the levels of PC (O-32:1) and LPC (20:3) in spinal cord samples increased in the SNI group and decreased after PL treatment. As a group of bioactive lipids, glycerophospholipids (i.e., PC and PE) can form corresponding lysophospholipids (i.e., LPC and LPE) under the catalytic influence of phospholipase D and phospholipase A2 (PLA2) and under varying pathophysiological conditions (Liu et al., 2020). The above PCs presented in serum and the spinal cord may be closely related to NP development. However, the role of LPCs is complicated, with both pro-inflammatory and anti-inflammatory properties (Knuplez and Marsche, 2020). Both pro-inflammatory and anti-inflammatory cytokines have been proven to exert important roles in NP (Sommer et al., 2018). LPC (20:3) in the spinal cord is probably a pro-inflammatory lipid while LPCs in serum may exhibit anti-inflammatory effect. Cers are the breakdown products of SM, and Cers are reported to trigger strong proinflammatory response. Cer (d18:1/24:1) was observed to be increased in NP and could be attenuated by PLM and PLH. Therefore, we hypothesized that PL-mediated reversionary effects of LPC and Cers could provide a potential explanation for the previously reported relief of NP by PL through suppression of neuroinflammation. TGs were found to be the risk factor of chronic pain (Sibille et al., 2016). The elevated concentrations of TG (18:0/18:1/20:4) and TG (20:0/20:0/20:4) in the SNI group may be associated with the occurrence of NP and could be regulated by PL. Taken together, PL may ameliorate NP by targeting lipid metabolism.

To validate and further lend credence to the lipidomics findings, transcriptomics was performed. Since the transcriptome of any biological system is cell- or tissue-specific, and the development of NP requires mechanisms that extend from the periphery to the spinal cord (Cohen and Mao, 2014), our choice of sample provided the relevant information pertaining to the spinal cord site. GO enrichment analysis for biological process associated with lipid includes responses to lipid, cellular lipid metabolic process, and lipid metabolic process (Supplementary Table S3), which highlights the importance of lipid metabolism in NP treatment. Pathway analysis also indicated that the calcium signaling pathway may play a critical role in the treatment of NP with PL.

**FIGURE 6:**
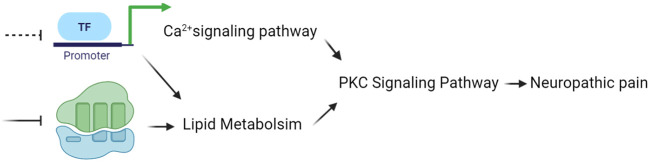
Working model of PL. PL may alleviate neuropathic pain by coordinating the calcium signaling and lipid metabolism to suppress PKC signaling transduction. PL, paeoniflorin and liquiritin combination.

Integrated analysis may provide a better understanding of PL therapy. Lipidomics and transcriptomics analysis revealed that lipid metabolism was of greatest impact and the calcium signaling pathway was most significant. Ca2+ could interact with lipid metabolism in several different ways. Calcium homeostasis and lipid metabolism were associated in present knowledge since Ca2+ could be stored in the endoplasmic reticulum, which is also a large, dynamic structure for lipid metabolism (Schwarz and Blower, 2016; Szymański et al., 2017). As two key second messengers, Ca2+ and signaling lipids, although extremely different in nature, play critical and often synergistic roles in signaling cascades. PKCs are perfectly designed to accurately decode all the attributes of the second two messengers. PKCs can combine the ability to read-out simultaneous lipid and Ca2+ signals and then convey the signaling content to downstream processes (Lipp and Reither, 2011). The spinal cord is the first relay site of the central nervous system to transmit nociceptive information (Zhuo et al., 2011). When NP occurs, the activated presynaptic neurons release numerous excitatory neurotransmitters to bind with specific receptors on the postsynaptic membrane. Stimulated membrane-bound receptors trigger intracellular Ca2+ influx and activate intracellular Ca2+-dependent downstream signal transduction pathways. In case of Gi protein-coupled receptors, following activation and dissociation of Gαβγ protein, Gβγ subunits can activate PLC which hydrolyses phosphatidylinositol-4,5-bisphosphate to inositol 1,4,5-trisphosphate (IP3) (Celik et al., 2016). Consequently, IP3 activates IP3R in the endoplasmic reticulum to mobilize intracellular Ca2+ (Samways and Henderson, 2006). In addition to IP3, PLC also generates diacylglycerol, diacylglycerol, and/or Ca2+ activate PKC, which can promote the activation of transcription factors, such as CREB, to further enhance pain transmission (Bao et al., 2021). Therefore, PL may alleviate NP by coordinating the calcium signaling and lipid metabolism to suppress PKC signaling transduction (Figure 6). Our results suggest that lipid and transcriptional changes provided useful information to better understand the therapeutic effect and mechanism of PL for NP.

## Conclusion

This study demonstrated the effect of PL against NP. Integrated lipidomics and transcriptomics revealed that anti-NP may be related to sphingolipid metabolism, glycerophospholipid metabolism, glycerolipid metabolism, and the calcium signaling pathway. Lipid metabolism and calcium signaling coordination may account for the major mechanism of PL for NP treatment. Altogether, this work provided new insights into the mechanism of PL in NP treatment, and suggested that the lipidomics and transcriptomics integration may be helpful to clarify the drug effect on NP.

## Data Availability

The datasets presented in this study can be found in online repositories. The names of the repository/repositories and accession number(s) can be found below: NCBI GEO, GSE205906.

## References

[B1] Bao Y. N. Dai W. L. Fan J. F. Ma B. Li S. S. Zhao W. L. (2021). The dopamine D1-d2dr complex in the rat spinal cord promotes neuropathic pain by increasing neuronal excitability after chronic constriction injury. Exp. Mol. Med. 53, 235–249. 10.1038/s12276-021-00563-5 33558591 PMC8080784

[B2] Celik M. Labuz D. Henning K. Busch-Dienstfertig M. Gaveriaux-Ruff C. Kieffer B. L. (2016). Leukocyte opioid receptors mediate analgesia via Ca(2+)-regulated release of opioid peptides. Brain Behav. Immun. 57, 227–242. 10.1016/j.bbi.2016.04.018 27139929

[B3] Chaparro L. E. Wiffen P. J. Moore R. A. Gilron I. (2012). Combination pharmacotherapy for the treatment of neuropathic pain in adults. Cochrane Database Syst. Rev. 2012, Cd008943. 10.1002/14651858.CD008943.pub2 PMC6481651 22786518

[B4] Chaplan S. R. Bach F. W. Pogrel J. W. Chung J. M. Yaksh T. L. (1994). Quantitative assessment of tactile allodynia in the rat paw. J. Neurosci. Methods 53, 55–63. 10.1016/0165-0270(94)90144-9 7990513

[B5] Cohen S. P. Mao J. (2014). Neuropathic pain: Mechanisms and their clinical implications. Bmj 348, F7656. 10.1136/bmj.f7656 24500412

[B6] Colloca L. Ludman T. Bouhassira D. Baron R. Dickenson A. H. Yarnitsky D. (2017). Neuropathic pain. Nat. Rev. Dis. Prim. 3, 17002. 10.1038/nrdp.2017.2 28205574 PMC5371025

[B7] Decosterd I. Woolf C. J. (2000). Spared nerve injury: An animal model of persistent peripheral neuropathic pain. Pain 87, 149–158. 10.1016/S0304-3959(00)00276-1 10924808

[B8] Echeverry S. Shi X. Q. Yang M. Huang H. Wu Y. Lorenzo L. E. (2017). Spinal microglia are required for long-term maintenance of neuropathic pain. Pain 158, 1792–1801. 10.1097/j.pain.0000000000000982 28746078

[B9] Finnerup N. B. Kuner R. Jensen T. S. (2021). Neuropathic pain: From mechanisms to treatment. Physiol. Rev. 101, 259–301. 10.1152/physrev.00045.2019 32584191

[B10] Guo Q. Li W. Wang C. Mao X. Wang X. Chen W. (2020). Biomolecular network-based synergistic drug combination discovery: A combination of paeoniflorin and liquiritin alleviates neuropathic pain by inhibiting neuroinflammation via suppressing the chemokine signaling pathway. Signal Transduct. Target. Ther. 5, 73. 10.1038/s41392-020-0160-8 32439892 PMC7242454

[B11] Hu B. Xu G. Zhang X. Xu L. Zhou H. Ma Z. (2018). Paeoniflorin attenuates inflammatory pain by inhibiting microglial activation and akt-NF-κb signaling in the central nervous system. Cell. Physiol. biochem. 47, 842–850. 10.1159/000490076 29807368

[B12] Karczewski K. J. Snyder M. P. (2018). Integrative omics for health and disease. Nat. Rev. Genet. 19, 299–310. 10.1038/nrg.2018.4 29479082 PMC5990367

[B13] Knuplez E. Marsche G. (2020). An updated review of pro- and anti-inflammatory properties of plasma lysophosphatidylcholines in the vascular system. Int. J. Mol. Sci. 21, E4501. 10.3390/ijms21124501 32599910 PMC7350010

[B14] Langeslag M. Kress M. (2020). The ceramide-S1p pathway as A druggable target to alleviate peripheral neuropathic pain. Expert Opin. Ther. Targets 24, 869–884. 10.1080/14728222.2020.1787989 32589067

[B15] Lipp P. Reither G. (2011). Protein kinase C: The "masters" of calcium and lipid. Cold Spring Harb. Perspect. Biol. 3, a004556. 10.1101/cshperspect.a004556 21628429 PMC3119906

[B16] Liu P. Zhu W. Chen C. Yan B. Zhu L. Chen X. (2020). The mechanisms of lysophosphatidylcholine in the development of diseases. Life Sci. 247, 117443. 10.1016/j.lfs.2020.117443 32084434

[B17] Mihara Y. Horikawa M. Sato S. Eto F. Hanada M. Banno T. (2019). Lysophosphatidic acid precursor levels decrease and an arachidonic acid-containing phosphatidylcholine level increases in the dorsal root ganglion of mice after peripheral nerve injury. Neurosci. Lett. 698, 69–75. 10.1016/j.neulet.2018.12.035 30593874

[B18] Patti G. J. Yanes O. Shriver L. P. Courade J. P. Tautenhahn R. Manchester M. (2012). Metabolomics implicates altered sphingolipids in chronic pain of neuropathic origin. Nat. Chem. Biol. 8, 232–234. 10.1038/nchembio.767 22267119 PMC3567618

[B19] Rimola V. Hahnefeld L. Zhao J. Jiang C. Angioni C. Schreiber Y. (2020). Lysophospholipids contribute to oxaliplatin-induced acute peripheral pain. J. Neurosci. 40, 9519–9532. 10.1523/JNEUROSCI.1223-20.2020 33158961 PMC7724144

[B20] Samways D. S. Henderson G. (2006). Opioid elevation of intracellular free calcium: Possible mechanisms and physiological relevance. Cell. Signal. 18, 151–161. 10.1016/j.cellsig.2005.08.005 16199136

[B21] Schwarz D. S. Blower M. D. (2016). The endoplasmic reticulum: Structure, function and response to cellular signaling. Cell. Mol. Life Sci. 73, 79–94. 10.1007/s00018-015-2052-6 26433683 PMC4700099

[B22] Sibille K. T. Steingrímsdóttir Ó A. Fillingim R. B. Stubhaug A. Schirmer H. Chen H. (2016). Pain Res. Manag. 2016, 7657329. 10.1155/2016/7657329Investigating The Burden Of Chronic Pain: An Inflammatory And Metabolic Composite 27445627 PMC4909918

[B23] Sommer C. Leinders M. Üçeyler N. (2018). Inflammation in the pathophysiology of neuropathic pain. Pain 159, 595–602. 10.1097/j.pain.0000000000001122 29447138

[B24] Szymański J. Janikiewicz J. Michalska B. Patalas-Krawczyk P. Perrone M. Ziółkowski W. (2017). Interaction of mitochondria with the endoplasmic reticulum and plasma membrane in calcium homeostasis, lipid trafficking and mitochondrial structure. Int. J. Mol. Sci. 18, E1576. 10.3390/ijms18071576 28726733 PMC5536064

[B25] Yang K. Han X. (2016). Lipidomics: Techniques, applications, and outcomes related to biomedical sciences. Trends biochem. Sci. 41, 954–969. 10.1016/j.tibs.2016.08.010 27663237 PMC5085849

[B26] Yin D. Liu Y. Y. Wang T. X. Hu Z. Z. Qu W. M. Chen J. F. (2016). Paeoniflorin exerts analgesic and hypnotic effects via adenosine A1 receptors in A mouse neuropathic pain model. Psychopharmacol. Berl. 233, 281–293. 10.1007/s00213-015-4108-6 26514553

[B27] Zhang M. T. Wang B. Jia Y. N. Liu N. Ma P. S. Gong S. S. (2017). Neuroprotective effect of liquiritin against neuropathic pain induced by chronic constriction injury of the sciatic nerve in mice. Biomed. Pharmacother. 95, 186–198. 10.1016/j.biopha.2017.07.167 28843150

[B28] Zhou D. Zhang S. Hu L. Gu Y. F. Cai Y. Wu D. (2019). Inhibition of apoptosis signal-regulating kinase by paeoniflorin attenuates neuroinflammation and ameliorates neuropathic pain. J. Neuroinflammation 16, 83. 10.1186/s12974-019-1476-6 30975172 PMC6458750

[B29] Zhuo M. Wu G. Wu L. J. (2011). Neuronal and microglial mechanisms of neuropathic pain. Mol. Brain 4, 31. 10.1186/1756-6606-4-31 21801430 PMC3163530

