# Supplementary material for: Combination of paeoniflorin and liquiritin alleviates neuropathic pain by lipid metabolism and calcium signaling coordination
Source: Front Pharmacol. 2022 Sep 7;13:944386. doi: 10.3389/fphar.2022.944386 (PMC9489943; doi:10.3389/fphar.2022.944386)
Supplement: Supplementary file 1 [file DataSheet1.DOCX]

Supplementary Material

# Supplementary Figures and Tables

## Supplementary Figures

**Supplementary Figure 1.** OPLS-DA plots of JA group and SNI group in serum samples (A) and tissue samples (B). Validation plots for OPLS-DA model in serum samples (C) and tissue samples (D). JA: sham operated group, SNI: spared nerve injury group.

## Supplementary Tables

**Supplementary Table 1.** The information of the identified lipids in the biological samples for constructing the database on the LC-MS platform

| Lipids | Retention Time (min) | Parent ion | Daughter ion | Lipids | Retention Time (min) | Parent ion | Daughter ion |
| --- | --- | --- | --- | --- | --- | --- | --- |
| PC(14:0/16:0) | 6.7867 | 706.5392 | 184.0739 | PE(18:0/20:4) | 8.2068 | 768.5549 | 627.5347 |
| PC(15:0/16:0) | 7.1998 | 720.5548 | 184.0739 | PE(18:1/20:4) | 7.2633 | 766.5392 | 625.5191 |
| PC(16:0/16:0) | 7.6456 | 734.5705 | 184.0739 | PE(18:2/20:4) | 7.0220 | 764.5236 | 623.5034 |
| PC(16:0/16:1) | 7.0601 | 732.5548 | 184.0739 | PE(18:2/22:2) | 8.6966 | 796.5862 | 655.566 |
| PC(16:1/16:1) | 6.2145 | 730.5392 | 184.0739 | PE(18:0/22:5) | 8.1433 | 794.5705 | 653.5504 |
| PC(15:0/18:0) | 8.0671 | 748.5861 | 184.0739 | PE(18:0/22:6) | 7.9147 | 792.5549 | 651.5347 |
| PC(15:0/18:1) | 7.4030 | 746.5705 | 184.0739 | PE(18:1/22:6) | 7.1871 | 790.5392 | 649.5191 |
| PC(15:0/18:2) | 6.6905 | 744.5548 | 184.0739 | PE(O-16:0/20:2) | 8.7999 | 730.5746 | 589.5545 |
| PC(16:0/18:0) | 8.4731 | 762.6018 | 184.0739 | PE(O-18:1/18:2) | 8.7569 | 728.559 | 392 |
| PC(16:0/18:1) | 7.8361 | 760.5861 | 184.0739 | PE(O-18:2/18:2) | 7.8996 | 726.5433 | 585.5232 |
| PC(16:0/18:2) | 7.1363 | 758.5705 | 184.0739 | PE(O-16:1/20:4) | 7.8361 | 724.5277 | 364 |
| PC(16:1/18:2) | 6.4019 | 756.5548 | 184.0739 | PE(O-20:1/18:3) | 8.6881 | 752.559 | 392 |
| PC(16:1/18:3) | 6.1512 | 754.5392 | 184.0739 | PE(O-20:4/18:2) | 7.5821 | 750.5433 | 609.5232 |
| PC(18:3/16:2) | 5.4412 | 752.5235 | 184.0739 | PE(O-18:2/20:4) | 7.9504 | 750.5433 | 390 |
| PC(17:0/18:0) | 8.8601 | 776.6174 | 184.0739 | PE(O-16:1/22:6) | 7.5186 | 748.5277 | 364 |
| PC(17:0/18:1) | 8.2449 | 774.6018 | 184.0739 | PE(O-18:3/20:4) | 7.2252 | 748.5277 | 388 |
| PC(17:0/18:2) | 7.5948 | 772.5861 | 184.0739 | PE(O-18:0/22:5) | 9.1553 | 780.5903 | 392 |
| PC(17:1/18:2) | 6.8823 | 770.5705 | 184.0739 | PE(O-20:1/20:5) | 8.6278 | 778.5746 | 392 |
| PC(17:1/18:3) | 6.6314 | 768.5548 | 184.0739 | PE(O-18:1/22:6) | 8.3846 | 776.559 | 392 |
| PC(18:0/18:0) | 9.2241 | 790.6331 | 184.0739 | PE(O-18:2/22:6) | 7.6710 | 774.5436 | 390 |
| PC(18:0/18:1) | 8.6622 | 788.6174 | 184.0739 | LPE 16:0 | 1.8497 | 454.2939 | 313.2738 |
| PC(18:0/18:2) | 8.0290 | 786.6018 | 184.0739 | LPE 18:0 | 2.3857 | 482.3252 | 341.3051 |
| PC(18:0/18:3) | 7.3141 | 784.5861 | 184.0739 | LPE 18:1 | 1.9527 | 480.3096 | 339.2894 |
| PC(18:2/18:2) | 7.0855 | 782.5705 | 184.0739 | LPE 18:2 | 1.6540 | 478.2939 | 337.2738 |
| PC(16:0/20:5) | 6.4426 | 780.5548 | 184.0739 | Cer(d18:1/16:0) | 7.8742 | 538.5194 | 264.2635 |
| PC(16:1/20:5) | 5.8745 | 778.5392 | 184.0739 | Cer(d18:1/20:0) | 9.4735 | 594.582 | 264.2635 |
| PC(15:0/22:0) | 9.8545 | 804.6487 | 184.0739 | Cer(d18:1/22:0) | 10.2415 | 622.6133 | 264.2635 |
| PC(15:0/22:2) | 8.4354 | 800.6174 | 184.0739 | Cer(d18:1/22:2) | 10.5769 | 618.582 | 264.2635 |
| PC(15:0/22:3) | 7.8361 | 798.6018 | 184.0739 | Cer(d18:1/24:0) | 10.8994 | 650.6446 | 264.2635 |
| PC(15:0/22:4) | 7.5440 | 796.5861 | 184.0739 | Cer(d18:1/24:1) | 10.2028 | 648.6289 | 264.2635 |
| PC(15:0/22:6) | 6.3464 | 792.5548 | 184.0739 | Cer(d18:2/24:0) | 10.4006 | 648.6289 | 262.2479 |
| PC(18:0/20:0) | 10.2028 | 818.6644 | 184.0739 | Hex2Cer(d18:1/24:0) | 10.4006 | 812.6975 | 264.2635 |
| PC(18:0/20:1) | 9.4219 | 816.6487 | 184.0739 | SM(d16:1/12:0) | 3.9257 | 619.482 | 184.0739 |
| PC(18:0/20:2) | 8.6795 | 814.6331 | 184.0739 | SM(d18:1/13:0) | 5.3779 | 661.5289 | 184.0739 |
| PC(18:0/20:3) | 8.2449 | 812.6174 | 184.0739 | SM(d18:1/14:0) | 5.8345 | 675.5446 | 184.0739 |
| PC(18:0/20:4) | 7.9655 | 810.6018 | 184.0739 | SM(d18:2/14:0) | 5.0045 | 673.5289 | 184.0739 |
| PC(18:1/20:4) | 7.1998 | 808.5861 | 184.0739 | SM(d18:0/15:0) | 6.8912 | 691.5759 | 184.0739 |
| PC(18:0/20:5) | 7.0474 | 808.5861 | 184.0739 | SM(d18:1/15:0) | 6.3479 | 689.5602 | 184.0739 |
| PC(18:2/20:3) | 6.7934 | 808.5861 | 184.0739 | SM(d18:2/15:0) | 5.4612 | 687.5446 | 184.0739 |
| PC(18:2/20:4) | 6.7979 | 806.5705 | 184.0739 | SM(d18:0/16:0) | 6.8275 | 705.5915 | 184.0739 |
| PC(16:0/22:6) | 6.5241 | 806.5705 | 184.0739 | SM(d18:1/16:0) | 6.8348 | 703.5759 | 184.0739 |
| PC(16:1/22:7) | 5.8045 | 804.5548 | 184.0739 | SM(d18:2/16:0) | 5.9912 | 701.5602 | 184.0739 |
| PC(17:0/22:2) | 9.2155 | 828.6487 | 184.0739 | SM(d18:0/17:0) | 7.6583 | 719.6072 | 184.0739 |
| PC(17:0/22:3) | 8.6537 | 826.6331 | 184.0739 | SM(d18:1/17:0) | 7.3141 | 717.5915 | 184.0739 |
| PC(17:0/22:4) | 8.3973 | 824.6174 | 184.0739 | SM(d18:2/17:0) | 6.5345 | 715.5759 | 184.0739 |
| PC(17:0/22:5) | 7.6329 | 822.6018 | 184.0739 | SM(d18:1/18:0) | 7.7599 | 731.6072 | 184.0739 |
| PC(17:0/22:6) | 7.2633 | 820.5861 | 184.0739 | SM(d18:1/18:1) | 7.0474 | 729.5915 | 184.0739 |
| PC(20:0/20:0) | 10.6113 | 846.6957 | 184.0739 | SM(d18:2/18:1) | 6.3079 | 727.5759 | 184.0739 |
| PC(20:0/20:1) | 10.1082 | 844.68 | 184.0739 | SM(d18:2/18:2) | 5.4645 | 725.5602 | 184.0739 |
| PC(22:0/18:1) | 9.9405 | 844.68 | 184.0739 | SM(d18:1/19:0) | 7.6988 | 745.6228 | 184.0739 |
| PC(20:0/20:2) | 9.6025 | 842.6644 | 184.0739 | SM(d18:2/19:0) | 7.6316 | 743.6072 | 184.0739 |
| PC(20:0/20:3) | 9.0435 | 840.6487 | 184.0739 | SM(d18:1/20:0) | 8.6622 | 759.6385 | 184.0739 |
| PC(20:0/20:4) | 8.4988 | 838.6331 | 184.0739 | SM(d18:2/20:0) | 7.9758 | 757.6228 | 184.0739 |
| PC(18:0/22:4) | 8.2667 | 838.6331 | 184.0739 | SM(d18:2/20:1) | 7.1490 | 755.6072 | 184.0739 |
| PC(20:0/20:5) | 7.9274 | 836.6174 | 184.0739 | SM(d19:1/20:0) | 9.0693 | 773.6541 | 184.0739 |
| PC(18:0/22:5) | 7.6861 | 836.6174 | 184.0739 | SM(d19:1/20:1) | 8.4227 | 771.6385 | 184.0739 |
| PC(18:0/22:6) | 7.6837 | 834.6018 | 184.0739 | SM(d18:1/22:0) | 9.4735 | 787.6698 | 184.0739 |
| PC(18:1/22:6) | 6.8950 | 832.5861 | 184.0739 | SM(d18:2/22:0) | 8.6795 | 785.6541 | 184.0739 |
| PC(18:2/22:6) | 6.4093 | 830.5705 | 184.0739 | SM(d18:2/22:1) | 7.0892 | 783.6385 | 184.0739 |
| PC(19:0/22:2) | 9.9491 | 856.68 | 184.0739 | SM(d18:3/22:2) | 6.9458 | 779.6072 | 184.0739 |
| PC(19:0/22:5) | 8.3338 | 850.6331 | 184.0739 | SM(d18:0/23:0) | 9.8502 | 803.7011 | 184.0739 |
| PC(19:0/22:6) | 8.1179 | 848.6174 | 184.0739 | SM(d18:1/23:0) | 9.8545 | 801.6854 | 184.0739 |
| PC(19:1/22:6) | 7.3268 | 846.6018 | 184.0739 | SM(d18:2/23:0) | 9.2757 | 799.6698 | 184.0739 |
| PC(18:0/24:1) | 10.7575 | 872.7113 | 184.0739 | SM(d18:0/24:0) | 10.2114 | 817.7167 | 184.0739 |
| PC(20:2/22:0) | 10.2888 | 870.6957 | 184.0739 | SM(d18:1/24:0) | 10.2114 | 815.7011 | 184.0739 |
| PC(18:2/24:1) | 9.5681 | 868.68 | 184.0739 | SM(d18:2/24:0) | 9.6627 | 813.6854 | 184.0739 |
| PC(18:2/24:2) | 9.0349 | 866.6644 | 184.0739 | SM(d18:2/24:2) | 8.1179 | 809.6541 | 184.0739 |
| PC(18:2/24:3) | 8.7913 | 864.6487 | 184.0739 | SM(d18:2/24:3) | 7.6456 | 807.6385 | 184.0739 |
| PC(18:3/24:2) | 8.5332 | 864.6487 | 184.0739 | SM(d18:2/24:4) | 7.0093 | 805.6228 | 184.0739 |
| PC(20:4/22:2) | 8.1941 | 862.6331 | 184.0739 | SM(d18:2/24:5) | 6.5907 | 803.6072 | 184.0739 |
| PC(20:5/22:2) | 7.7472 | 860.6174 | 184.0739 | SM(d18:1/25:0) | 10.4651 | 829.7167 | 184.0739 |
| PC(20:4/22:4) | 7.0601 | 858.6018 | 184.0739 | SM(d18:2/25:0) | 9.7857 | 827.7011 | 184.0739 |
| PC(20:4/22:5) | 6.4131 | 856.5861 | 184.0739 | SM(d18:0/26:1) | 10.8736 | 843.7324 | 184.0739 |
| PC(20:4/22:6) | 6.1207 | 854.5705 | 184.0739 | SM(d18:1/26:1) | 10.1727 | 841.7167 | 184.0739 |
| PC(22:2/22:0) | 10.8908 | 898.727 | 184.0739 | SM(d18:1/26:2) | 9.6369 | 839.7011 | 184.0739 |
| PC(O-14:0/16:0) | 7.3395 | 692.5588 | 184.0739 | TG(16:0/14:0/16:0) | 12.6354 | 796.7429 | 523.4734 |
| PC(O-14:0/16:1) | 6.8950 | 690.5432 | 184.0739 | TG(16:1/14:0/18:2) | 11.9396 | 818.7229 | 521.457 |
| PC(O-15:0/16:1) | 7.7726 | 706.5745 | 184.0739 | TG(16:1/14:0/18:1) | 12.4093 | 820.7394 | 521.4563 |
| PC(O-16:0/16:0) | 8.2068 | 720.5902 | 184.0739 | TG(16:0/14:0/18:1) | 12.6823 | 822.7543 | 523.47 |
| PC(O-16:0/16:1) | 7.6583 | 718.5745 | 184.0739 | TG(16:1/15:0/18:2) | 12.3608 | 832.7394 | 535.4721 |
| PC(O-16:1/16:0) | 8.1536 | 718.5745 | 184.0739 | TG(16:0/15:0/18:2) | 12.5262 | 834.7551 | 561.49 |
| PC(O-16:0/16:2) | 7.5821 | 716.5589 | 184.0739 | TG(16:0/15:1/18:1) | 12.6354 | 834.7598 | 535.47 |
| PC(O-16:0/18:0) | 9.0005 | 748.6214 | 184.0739 | TG(18:0p/16:0/16:0) | 12.8891 | 836.8049 | 563.54 |
| PC(O-16:0/18:1) | 8.3211 | 746.6058 | 184.0739 | TG(16:0/16:0/17:0) | 13.2119 | 838.787 | 565.52 |
| PC(O-16:0/18:2) | 7.6964 | 744.5902 | 184.0739 | TG(16:0/16:1/18:3) | 12.0487 | 844.7401 | 571.4768 |
| PC(O-16:1/18:1) | 8.3187 | 744.5902 | 184.0739 | TG(16:0/14:0/20:4) | 12.2298 | 844.7399 | 523.7399 |
| PC(O-16:0/18:3) | 7.6456 | 742.5745 | 184.0739 | TG(16:0/16:1/18:2) | 12.3702 | 846.7579 | 549.488 |
| PC(O-18:0/17:1) | 7.8281 | 760.6215 | 184.0739 | TG(16:0/16:1/18:1) | 12.7368 | 848.7694 | 549.48 |
| PC(O-18:0/18:0) | 9.7513 | 776.6527 | 184.0739 | TG(16:0/16:0/18:1) | 13.0624 | 850.7878 | 577.5193 |
| PC(O-18:0/18:1) | 9.0779 | 774.6371 | 184.0739 | TG(18:0/16:0/16:0) | 13.3809 | 852.8019 | 579.5356 |
| PC(O-18:0/18:3) | 7.6515 | 770.6058 | 184.0739 | TG(15:1/18:2/18:2) | 11.8303 | 856.7379 | 559.4719 |
| PC(O-18:1/18:2) | 7.8321 | 770.6058 | 184.0739 | TG(15:0/18:2/18:2) | 12.2126 | 858.7595 | 561.4888 |
| PC(O-18:1/18:3) | 7.6583 | 768.5902 | 184.0739 | TG(15:0/18:1/18:2) | 12.5808 | 860.7678 | 601.52 |
| PC(O-18:2/18:3) | 7.0220 | 766.5745 | 184.0739 | TG(16:0/17:1/18:1) | 12.9129 | 862.7846 | 589.5199 |
| PC(O-18:3/18:2) | 6.8823 | 766.5745 | 184.0739 | TG(16:0/17:0/18:1) | 13.2574 | 864.8029 | 591.5355 |
| PC(O-18:0/20:0) | 10.4479 | 804.684 | 184.0739 | TG(18:0/16:0/17:0) | 13.5191 | 866.8185 | 565.522 |
| PC(O-18:0/20:3) | 8.7569 | 798.6371 | 184.0739 | TG(16:0/18:2/18:3) | 12.0566 | 870.7554 | 597.48 |
| PC(O-18:0/20:4) | 8.2065 | 796.6215 | 184.0739 | TG(16:0/16:0/20:5) | 12.3078 | 870.7554 | 551.503 |
| PC(O-18:1/20:3) | 8.4988 | 796.6215 | 184.0739 | TG(16:0/18:2/18:2) | 12.4248 | 872.7715 | 575.5 |
| PC(O-18:0/20:5) | 7.7599 | 794.6058 | 184.0739 | TG(16:0/16:0/20:4) | 12.6354 | 872.7715 | 551.51 |
| PC(O-18:1/20:4) | 7.5948 | 794.6058 | 184.0739 | TG(16:0/18:1/18:1) | 13.1079 | 876.7967 | 603.53 |
| PC(O-18:1/20:5) | 7.6837 | 792.5902 | 184.0739 | TG(18:0/16:0/18:1) | 13.4324 | 878.8103 | 577.5192 |
| PC(O-18:2/20:4) | 7.5186 | 792.5902 | 184.0739 | TG(16:0/17:0/19:1) | 13.4199 | 878.8103 | 605.8 |
| PC(O-18:2/20:5) | 7.2633 | 790.5746 | 184.0739 | TG(18:0/16:0/18:0) | 13.7124 | 880.8355 | 579.5352 |
| PC(O-18:1/22:0) | 10.4694 | 830.6997 | 184.0739 | TG(17:1/18:2/18:2) | 12.3235 | 884.7731 | 599.51 |
| PC(O-18:0/22:4) | 9.2929 | 824.6528 | 184.0739 | TG(17:1/18:1/18:2) | 12.6354 | 886.7901 | 601.52 |
| PC(O-18:0/22:5) | 9.2212 | 822.6371 | 184.0739 | TG(17:0/18:1/18:2) | 12.9714 | 888.7957 | 601.52 |
| PC(O-18:2/22:3) | 8.9289 | 822.6371 | 184.0739 | TG(16:0/16:1/22:6) | 11.9551 | 894.7545 | 621.49 |
| PC(O-18:1/22:5) | 8.2195 | 820.6215 | 184.0739 | TG(16:0/18:2/20:5) | 11.9629 | 894.7555 | 575.47 |
| PC(O-18:2/22:5) | 8.1306 | 818.6058 | 184.0739 | TG(18:1/18:2/18:3) | 12.1189 | 896.7703 | 597.49 |
| PC(O-18:2/22:6) | 7.3649 | 816.5902 | 184.0739 | TG(16:0/16:0/22:6) | 12.3235 | 896.7714 | 623.51 |
| PC(O-20:0/22:4) | 10.0179 | 852.6841 | 184.0739 | TG(16:0/18:2/20:4) | 12.3156 | 896.7714 | 575.51 |
| PC(O-20:0/22:5) | 9.2671 | 850.6684 | 184.0739 | TG(18:2/18:2/18:2) | 12.1033 | 896.7714 | 599.5 |
| PC(O-20:0/22:6) | 8.6393 | 848.6528 | 184.0739 | TG(16:0/18:1/20:4) | 12.6823 | 898.7872 | 625.51 |
| PC(O-20:1/22:6) | 8.9375 | 846.6371 | 184.0739 | TG(18:1/18:2/18:2) | 12.4795 | 898.7872 | 601.5118 |
| PC(O-22:0/22:4) | 10.6672 | 880.7154 | 184.0739 | TG(18:1/18:1/18:1) | 13.1534 | 902.8176 | 603.535 |
| LPC 14:0 | 1.4480 | 468.3095 | 184.0739 | TG(18:0/18:1/18:2) | 13.1534 | 902.8176 | 601.52 |
| LPC 15:0 | 1.5819 | 482.3251 | 184.0739 | TG(18:0/18:1/18:1) | 13.4658 | 904.8345 | 603.53 |
| LPC 15:1 | 1.4377 | 480.3095 | 184.0739 | TG(18:0/18:0/18:1) | 13.7458 | 906.8462 | 605.54 |
| LPC 16:0 | 1.7776 | 496.3408 | 184.0739 | TG(18:1/18:2/19:1) | 13.0104 | 914.8185 | 615.54 |
| LPC 16:1 | 1.5201 | 494.3251 | 184.0739 | TG(18:1/18:1/19:1) | 13.3289 | 916.8314 | 617.8195 |
| LPC 16:2 | 1.3347 | 492.3095 | 184.0739 | TG(18:1/18:2/20:5) | 12.0331 | 920.771 | 621.49 |
| LPC 17:0 | 2.0042 | 510.3564 | 184.0739 | TG(16:0/18:2/22:6) | 12.1189 | 920.7716 | 647.51 |
| LPC 17:1 | 1.6952 | 508.3408 | 184.0739 | TG(18:1/18:2/20:4) | 12.4795 | 922.7871 | 623.5 |
| LPC 17:2 | 1.5201 | 506.3251 | 184.0739 | TG(16:0/18:1/22:6) | 12.2766 | 922.7876 | 649.52 |
| LPC 18:1 | 1.8600 | 522.3564 | 184.0739 | TG(16:0/18:2/22:5) | 12.1129 | 922.7876 | 575.51 |
| LPC 18:2 | 1.5922 | 520.3408 | 184.0739 | TG(18:0/18:1/20:4) | 13.0819 | 926.8185 | 605.54 |
| LPC 18:3 | 1.3965 | 518.3251 | 184.0739 | TG(16:0/18:1/22:4) | 12.9584 | 926.8185 | 577.51 |
| LPC 19:0 | 2.6257 | 538.3877 | 184.0739 | TG(18:0/18:1/20:3) | 13.2249 | 928.8366 | 605.54 |
| LPC 19:1 | 2.0866 | 536.3721 | 184.0739 | TG(20:1/18:1/18:2) | 13.1664 | 928.8366 | 601.524 |
| LPC 20:0 | 3.0757 | 552.4034 | 184.0739 | TG(20:1/18:1/18:1) | 13.4784 | 930.848 | 603.5346 |
| LPC 20:2 | 1.9527 | 548.3721 | 184.0739 | TG(16:0/18:1/22:1) | 13.7991 | 932.8657 | 659.5958 |
| LPC 20:3 | 1.7055 | 546.3564 | 184.0739 | TG(16:0/16:0/24:0) | 14.3691 | 936.8949 | 663.6283 |
| LPC 20:4 | 1.5613 | 544.3408 | 184.0739 | TG(18:1/18:2/22:6) | 12.1753 | 946.7888 | 647.51 |
| LPC 20:5 | 1.3759 | 542.3251 | 184.0739 | TG(18:0/20:4/20:5) | 12.3078 | 946.7888 | 645.486 |
| LPC 22:0 | 4.0724 | 580.4347 | 184.0739 | TG(18:1/18:1/22:6) | 12.5419 | 948.7911 | 649.52 |
| LPC 22:1 | 3.1657 | 578.419 | 184.0739 | TG(18:1/18:2/22:5) | 12.3390 | 948.7911 | 601.52 |
| LPC 22:3 | 2.1175 | 574.3877 | 184.0739 | TG(18:1/18:1/22:5) | 12.6823 | 950.8039 | 651.52 |
| LPC 22:5 | 1.5922 | 570.3564 | 184.0739 | TG(18:0/18:1/22:6) | 12.8739 | 950.8039 | 649.5186 |
| LPC 22:6 | 1.4995 | 568.3408 | 184.0739 | TG(20:1/18:1/20:4) | 13.0819 | 952.8363 | 625.526 |
| LPC 24:0 | 5.1712 | 608.466 | 184.0739 | TG(18:1/18:2/22:1) | 13.5044 | 956.8651 | 657.58 |
| LPC 24:1 | 4.1157 | 606.4503 | 184.0739 | TG(18:1/18:1/22:1) | 13.8624 | 958.8815 | 659.59 |
| LPC O-16:1 | 1.9733 | 480.3449 | 184.0739 | TG(16:0/18:1/24:1) | 14.1558 | 960.8967 | 687.63 |
| LPC O-18:1 | 2.0969 | 508.3762 | 184.0739 | TG(18:1/18:1/22:0) | 14.0791 | 960.8967 | 661.61 |
| LPC O-16:0 | 2.1896 | 534.3919 | 184.0739 | TG(16:0/18:1/24:0) | 14.3424 | 962.9099 | 689.64 |
| LPC O-20:0 | 1.9836 | 482.3605 | 184.0739 | TG(18:0/16:0/24:0) | 14.6424 | 964.9264 | 663.6283 |
| LPC O-20:2 | 2.6257 | 538.4231 | 184.0739 | TG(20:0/20:4/20:4) | 12.9259 | 976.8309 | 655.565 |
| PE(16:0/18:0) | 8.6881 | 720.5549 | 579.5347 | TG(24:1/18:2/18:2) | 13.5691 | 982.8775 | 599.5 |
| PE(16:0/18:1) | 7.9782 | 718.5392 | 577.5191 | TG(18:1/18:2/24:1) | 13.8424 | 984.8951 | 685.611 |
| PE(16:0/18:2) | 7.3903 | 716.5236 | 575.5034 | TG(18:1/18:1/24:1) | 14.1858 | 986.9093 | 687.63 |
| PE(17:0/18:2) | 7.8107 | 730.5392 | 589.5191 | TG(18:1/18:1/24:0) | 14.3791 | 988.9256 | 689.64 |
| PE(18:0/18:1) | 8.8514 | 746.5705 | 605.5504 | CE 18:2 | 12.6510 | 666.6183 | 369.3516 |
| PE(18:0/18:2) | 8.2449 | 744.5549 | 603.5347 | CE 18:3 | 12.2142 | 664.6027 | 369.3516 |
| PE(18:1/18:2) | 7.5313 | 742.5392 | 601.5191 | CE 20:4 | 12.3156 | 690.6183 | 369.3516 |
| PE(18:2/18:2) | 7.3141 | 740.5236 | 599.5034 | CE 22:6 | 12.0487 | 714.6183 | 369.3516 |
| PE(18:0/20:3) | 8.2150 | 770.5705 | 629.5504 |  |  |  |  |

**Supplementary Table 2.** Potential targets of paeoniflorin and liquiritin

| MyList | Gene ID | MyList | Gene ID | MyList | Gene ID | MyList6 | Gene ID |
| --- | --- | --- | --- | --- | --- | --- | --- |
| CYP19A1 | 1588 | PTPN2 | 5771 | CA7 | 766 | IRAK4 | 51135 |
| SLC5A1 | 6523 | ESR1 | 2099 | PLA2G1B | 5319 | FOLH1 | 2346 |
| SLC5A2 | 6524 | ESR2 | 2100 | PNP | 4860 | DHODH | 1723 |
| TYR | 7299 | MMP1 | 4312 | AMPD3 | 272 | LDHA | 3939 |
| SLC5A4 | 6527 | MMP7 | 4316 | PIM1 | 5292 | LDHB | 3945 |
| ADORA1 | 134 | MMP8 | 4317 | DYRK2 | 8445 | PLA2G2A | 5320 |
| EIF4A1 | 1973 | CA4 | 762 | RXRA | 6256 | PLA2G5 | 5322 |
| EPHX2 | 2053 | TERT | 7015 | HCAR2 | 338442 | PLA2G10 | 8399 |
| SLC28A3 | 64078 | SLC29A1 | 2030 | RARS | 5917 | YARS | 8565 |
| PTGS1 | 5742 | AKR1B1 | 231 | PTPN1 | 5770 | PIK3CA | 5290 |
| MAOB | 4129 | ADORA2B | 136 | TYMS | 7298 | SLC10A2 | 6555 |
| CYP1B1 | 1545 | CA14 | 23632 | KDM3A | 55818 | CASP3 | 836 |
| ADORA3 | 140 | CA3 | 761 | AKR1B10 | 57016 | HRAS | 3265 |
| TAS2R31 | 259290 | CA6 | 765 | KDM4C | 23081 | CASP6 | 839 |
| ABCC1 | 4363 | CA13 | 377677 | AKR1C3 | 8644 | CASP7 | 840 |
| HSD17B1 | 3292 | CA5B | 11238 | PYGL | 5836 | CASP8 | 841 |
| SHBG | 6462 | ABCB1 | 5243 | TTR | 7276 | CASP1 | 834 |
| CBR1 | 873 | CA5A | 763 | IGFBP3 | 3486 | CASP2 | 835 |
| MMP13 | 4322 | CA2 | 760 | SERPINE1 | 5054 | CHIA | 27159 |
| MMP12 | 4321 | CA1 | 759 | GRM5 | 2915 | PARP1 | 142 |
| ABCG2 | 9429 | AGTR1 | 185 | MAPK8 | 5599 | PRKACA | 5566 |
| CA12 | 771 | CES1 | 1066 | TYMP | 1890 | GART | 2618 |
| ADORA2A | 135 | CES2 | 8824 | BACE1 | 23621 | EGFR | 1956 |
| SRD5A1 | 6715 | ALB | 213 | MGMT | 4255 | PYGM | 5837 |
|  |  | SLC28A2 | 9153 | ATIC | 471 | PPARG | 5468 |

**Supplementary Table 3.** GO enrichment analysis table of biological process associated with lipid

| GO ID | Term Type | Description | Ratio_in_study | Ratio_in_pop | Pvalue | Padjust |
| --- | --- | --- | --- | --- | --- | --- |
| GO:0033993 | BP | response to lipid | 37/230 | 1342/24558 | 3.48E-09 | 2.65E-05 |
| GO:0044255 | BP | cellular lipid metabolic process | 23/230 | 999/24558 | 7.01E-05 | 0.016308 |
| GO:0006629 | BP | lipid metabolic process | 26/230 | 1256/24558 | 0.000205 | 0.035184 |
| GO:0034368 | BP | protein-lipid complex remodeling | 3/230 | 27/24558 | 0.002009 | 0.163529 |
| GO:0071396 | BP | cellular response to lipid | 14/230 | 671/24558 | 0.00619 | 0.31917 |
| GO:0071825 | BP | protein-lipid complex subunit organization | 3/230 | 43/24558 | 0.007595 | 0.361766 |
| GO:0008610 | BP | lipid biosynthetic process | 11/230 | 513/24558 | 0.009351 | 0.361766 |
| GO:0045834 | BP | positive regulation of lipid metabolic process | 6/230 | 195/24558 | 0.010341 | 0.380124 |
| GO:0046889 | BP | positive regulation of lipid biosynthetic process | 4/230 | 103/24558 | 0.016115 | 0.509271 |
| GO:0033700 | BP | phospholipid efflux | 2/230 | 21/24558 | 0.016313 | 0.509271 |
| GO:1902068 | BP | regulation of sphingolipid mediated signaling pathway | 1/230 | 2/24558 | 0.018644 | 0.510076 |
| GO:0046890 | BP | regulation of lipid biosynthetic process | 6/230 | 228/24558 | 0.0208 | 0.551197 |
| GO:0019216 | BP | regulation of lipid metabolic process | 9/230 | 431/24558 | 0.020845 | 0.551411 |
| GO:0006644 | BP | phospholipid metabolic process | 8/230 | 374/24558 | 0.025195 | 0.608803 |
| GO:0046486 | BP | glycerolipid metabolic process | 8/230 | 377/24558 | 0.026254 | 0.608803 |
| GO:1900130 | BP | regulation of lipid binding | 1/230 | 3/24558 | 0.027836 | 0.608803 |
| GO:1900131 | BP | negative regulation of lipid binding | 1/230 | 3/24558 | 0.027836 | 0.608803 |
| GO:0050994 | BP | regulation of lipid catabolic process | 3/230 | 72/24558 | 0.030146 | 0.639086 |
| GO:0050995 | BP | negative regulation of lipid catabolic process | 2/230 | 33/24558 | 0.038123 | 0.721155 |
| GO:0090218 | BP | positive regulation of lipid kinase activity | 2/230 | 36/24558 | 0.044669 | 0.782876 |
| GO:1903725 | BP | regulation of phospholipid metabolic process | 3/230 | 88/24558 | 0.04982 | 0.828765 |
